# Effect of tyrosine ingestion on cognitive function and load carriage performance in the heat

**DOI:** 10.1186/2046-7648-4-S1-A148

**Published:** 2015-09-14

**Authors:** Nicole Coull, Josh Foster, Bryna Chrismas, Lee Taylor

**Affiliations:** 1 Department of Sport Science and Physical Activity (SSPA), University of Bedfordshire, Bedford, UK

## Introduction

Prolonged exercise-heat-stress impairs both exercise performance and cognitive function. Military based operations are often performed in hot environmental conditions and thus performance and safety may be compromised which could be potentially life threatening. Ingestion of tyrosine (TYR), a catecholamine precursor has been shown to improve exercise performance [1] and cognitive function [2] in hot environments, but no study has assessed the effects of TYR in a simulated military setting in the heat simultaneously assessing steady sate exercise performance, time-trial performance and alterations in various facets of cognitive function. Therefore, the aim of this study was to investigate the effect of 150 mg.kg TYR on steady state exercise, cognitive function and time-trial performance in the heat.

## Methods

Eight recreationally active, healthy males [age 23(1) y, height 176.4(5.9) cm, body mass 79(11.5) kg] visited the laboratory on four occasions (two familiarisation and two experimental conditions). In a double-blind, counter-balanced, crossover design participants ingested a placebo [PLA (250 mL sugar free squash)] or tyrosine [TYR (same as PLA plus 150 mg.kg TYR powder)] 1 h pre-exercise. Participants completed a 60 min walk at 6.5 km.h^-1^, followed by a 2.4 km time-trial carrying a 25 kg backpack in 40 °C and 30 % rh. Aspects of cognitive function were assessed using the PsychE software package, including number vigilance (identification of a duplicate number), dual-task (tracking and stimuli response) and simple reaction time (stimuli response - thinking and movement time) at 5 time-points; pre-ingestion, pre-exercise, 30 min into exercise, post 60 min exercise and post time-trial. Measures of heart rate (HR), rating of perceived exertion (RPE), thermal sensation (TSS) and rectal (*T*_re_) and skin temperature (T_sk_) were recorded throughout the exercise and rest period.

## Results

A significant increase from pre-exercise to post 60 min exercise (*p *< 0.01) was observed for vigilance and dual-task FALSE scores, and for reaction time in both conditions. However, no significant difference was observed between TYR and PLA conditions in any of the cognitive tests measured (*p *> 0.05). Furthermore, no significant difference was observed in time-trial completion time (*F*_1,14 _= 547.9, *p *= 0.74) between TYR [19.78(3.44) min] and PLA [20.29(3.55) min]. No significant differences were observed in any of the physiological (HR), perceptual (RPE, TSS) or temperature measures between conditions (*p *> 0.05).

## Discussion

During TYR and PLA conditions, vigilance, dual task and reaction time cognitive processes declined pre to post exercise. This is surprising since increasing the provision of TYR (a catecholamine precursor), through oral ingestion is suggested to maintain catecholamine synthesis and thus alleviate stress-related decrements in performance; as shown elsewhere during soccer specific exercise [2].

## Conclusion

Ingestion of 150 mg.kg TYR did not influence cognitive function or any outcome variable associated with steady state exercise or time-trial performance after load carriage (25 kg) in a hot environment (40 °C).
